# Myoelectric Control in Rehabilitative and Assistive Soft Exoskeletons: A Comprehensive Review of Trends, Challenges, and Integration with Soft Robotic Devices

**DOI:** 10.3390/biomimetics10040214

**Published:** 2025-04-01

**Authors:** Alejandro Toro-Ossaba, Juan C. Tejada, Daniel Sanin-Villa

**Affiliations:** 1 Artificial Intelligence and Robotics Research Group (IAR), Universidad EIA, Envigado 055428, Colombia; alejandro.toro52@eia.edu.co; 2 Department of Engineering Studies for Innovation, Universidad Iberoamericana Ciudad de México, Prolongación Paseo de la Reforma 880, Colonia Lomas de Santa Fé, Ciudad de México 01219, Mexico; 3 Área Industria, Materiales y Energía, Universidad EAFIT, Medellín 050022, Colombia

**Keywords:** soft robotics, exoskeletons, myoelectric control, electromyography, motion intention estimation

## Abstract

Soft robotic exoskeletons have emerged as a transformative solution for rehabilitation and assistance, offering greater adaptability and comfort than rigid designs. Myoelectric control, based on electromyography (EMG) signals, plays a key role in enabling intuitive and adaptive interaction between the user and the exoskeleton. This review analyzes recent advancements in myoelectric control strategies, emphasizing their integration into soft robotic exoskeletons. Unlike previous studies, this work highlights the unique challenges posed by the deformability and compliance of soft structures, requiring novel approaches to motion intention estimation and control. Key contributions include critically evaluating machine learning-based motion prediction, model-free adaptive control methods, and real-time validation strategies to enhance rehabilitation outcomes. Additionally, we identify persistent challenges such as EMG signal variability, computational complexity, and the real-time adaptability of control algorithms, which limit clinical implementation. By interpreting recent trends, this review highlights the need for improved EMG acquisition techniques, robust adaptive control frameworks, and enhanced real-time learning to optimize human-exoskeleton interaction. Beyond summarizing the state of the art, this work provides an in-depth discussion of how myoelectric control can advance rehabilitation by ensuring more responsive and personalized exoskeleton assistance. Future research should focus on refining control schemes tailored to soft robotic architectures, ensuring seamless integration into rehabilitation protocols. This review is a foundation for developing intelligent soft exoskeletons that effectively support motor recovery and assistive applications.

## 1. Introduction

At present, there is growing research interest in robotic devices for rehabilitation and assistance, especially for exoskeletons. This interest stems from their usefulness in tasks such as enhancing user strength, supporting rehabilitation sessions, and aiding movements in cases where users have lost motor skills due to accidents or illnesses [1,2,3]. These devices offer the potential for more repeatable and efficient rehabilitation therapies or assistance movements while increasing adaptability and maintaining comfort for various users [4,5,6,7].

To enhance the adaptability and comfort of exoskeletons, designs based on soft robotics, which use deformable and soft materials, have been gaining interest. These materials can potentially adapt to the human body’s contours, providing greater comfort and performance in rehabilitation and assistance tasks [8,9,10,11]. However, this design trend has also introduced new challenges in developing and controlling rehabilitation and assistance exoskeletons.

While myoelectric control has been extensively studied in rigid exoskeletons, its implementation in soft exoskeletons presents additional challenges due to the deformable nature of these devices. The interaction between the soft material and the user’s anatomical structure introduces variations in force transmission, requiring advanced motion intention estimation and adaptive control strategies to ensure precise and ergonomic assistance. One key challenge in implementing soft rehabilitation and assistance exoskeletons is designing control schemes that enable intuitive, active, and semi-autonomous device control. Control schemes based on electrophysiological signals have shown promising results in addressing this challenge, with myoelectric control being widely studied for controlling active exoskeletons [12,13]. Other control strategies, such as electroencephalography (EEG) [14,15], mechanomyography (MMG) [16,17], and force myography (FMG) [18,19,20], have also been proposed. However, electromyography (EMG) has proven to be the most feasible alternative for intuitively controlling exoskeletons [21]. Despite the extensive study of myoelectric control based on EMG, some drawbacks still limit its commercial application [13,22,23,24].

This article comprehensively reviews the current state of soft robotic exoskeletons for rehabilitation and assistance. In particular, it focuses on the state-of-the-art of myoelectric control strategies. This paper is organized as follows: Section 2 presents the methods used in this systemic review, including the search queries and the inclusion and exclusion criteria. Section 3 discusses the fundamental concepts of rehabilitation and assistance with soft robotic exoskeletons and presents the state-of-the-art in myoelectric control. Section 5 discusses the key findings of this review and the potential of using myoelectric control strategies in soft robotic exoskeletons. Section 4 presents the current challenges in myoelectric control. Finally, Section 6 presents the conclusions of this review.

## 2. Methodological Approach

This review examines the integration of myoelectric control strategies with soft robotics exoskeletons for rehabilitation and assistance, emphasizing their combined challenges and opportunities. To comprehensively cover the latest advancements, a systematic literature search was carried out using the SCOPUS and Web of Science databases, supplemented by tools like *Research Rabbit* and *Elicit* for data exploration and synthesis. These tools enabled iterative searches, relationship mapping, and the identification of relevant studies in this emerging interdisciplinary field.

### 2.1. Search Strategy

The search utilized structured queries that incorporated Boolean operators and keywords pertinent to the primary subjects of soft robotics, exoskeletons, and myoelectric control. The keywords were categorized into three distinct themes:

- Soft Robotics: “soft robotics”, “soft exoskeletons”, “control of soft robots”, “biomimetic robotics”.
- Exoskeletons: “rehabilitation and assistance exoskeletons”, “soft exoskeletons”, “elbow exoskeletons”, “control of exoskeletons”.
- Myoelectric Control: “myoelectric control”, “surface electromyography”, “sEMG control”, “motion intention estimation”, “sEMG joint torque estimation”, “sEMG joint position estimation”.

An example query used in SCOPUS was the following

TITLE-ABS-KEY(Control AND (electromyography OR “surface electromyography” OR myoelectric OR emg OR semg) AND (orthesis OR exoskeleton OR robot* OR assit*))

### 2.2. Inclusion and Exclusion Criteria

The selection process focused on peer-reviewed articles chosen for their relevance and significance in soft robotics, rehabilitation and assistance exoskeletons, and myoelectric control, irrespective of their publication year. the inclusion criteria included the following:

- Articles from 2012 to 2025 were included.
- Research involving systems for rehabilitation or assistance of the upper and lower limbs, primarily systems focused on the elbow or knee.
- Both rigid and soft systems were included, although the focus was on soft robotic exoskeletons.
- Articles that integrated control strategies with rehabilitation and assistance exoskeletons.
- Articles with knee systems were considered because the knee is a similar joint to the elbow, making some systems identical for both joints.
- Articles on the processing and selection of EMG signal features.

Articles were excluded if they met the following exclusion criteria:

- Articles that lacked full-text access or were not peer-reviewed.
- Articles that focused solely on hardware design.
- Articles focused on gesture recognition in the hand were excluded.

### 2.3. Data Filtering and Selection

The preliminary search yielded around 900 articles. After removing duplicates and an abstract relevance screening, 312 articles were reviewed in full-text. The final dataset comprised 150 articles, focusing on studies addressing soft robotics exoskeletons for rehabilitation and assistance, particularly emphasizing their integration with myoelectric control strategies.

**Figure 1:**
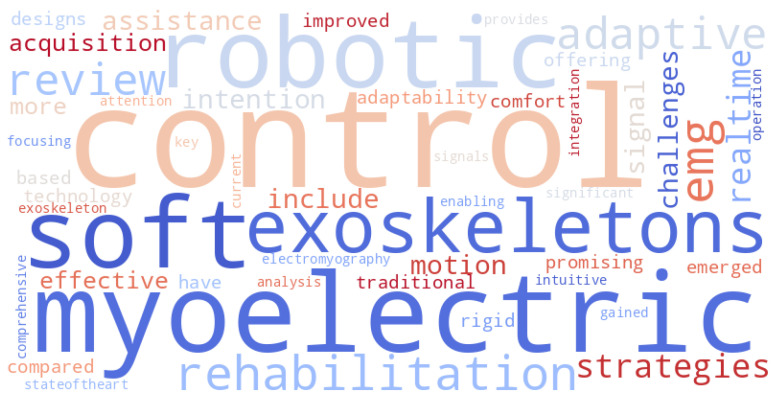
Word cloud summarizing the key themes and concepts explored in this review. The most frequent terms highlight key concepts related to soft robotic exoskeletons, myoelectric control, and rehabilitation strategies.

Figure 1 presents a word cloud generated from the main topics covered in this review. The most frequently occurring terms, such as “soft robotics”, “exoskeletons”, “myoelectric control”, and “rehabilitation”, highlight the key focus areas of this study. The prominence of terms related to control strategies, motion intention estimation, and electromyography (EMG) signal processing emphasizes the importance of integrating advanced myoelectric control techniques in soft robotic exoskeletons. This visualization provides an intuitive representation of the critical themes explored in this work.

## 3. Theoretical Framework and State-of-the-Art in Myoelectric Control Strategies for Soft Robotic Exoskeletons

### 3.1. Robotic Assistance and Rehabilitation Systems

The F48 ASTM committee on exoskeletons and exosuits describes an exoskeleton as a system that “augments, enables, assists, and/or enhances physical activity through mechanical interaction with the body and may include rigid or soft components, or both” [25]. Typically, robotic exoskeletons are designed for three primary applications: augmentation, assistance, and rehabilitation.

Augmentation applications involve robotic assistive devices that provide additional torque and support to enhance the user’s physical abilities. These devices help users lift or move heavier loads and redistribute the load at specific joints. The main goals are reducing metabolic costs, decreasing fatigue, and preventing musculoskeletal disorders caused by repetitive movements [26,27].

In assistance applications, robotic devices aim to support users with reduced limb capabilities due to musculoskeletal or neuromuscular conditions. These devices assist in performing activities of daily living (ADLs), thereby improving the user’s quality of life [26,28,29].

Rehabilitation therapies rely on repetitive movement execution to promote neuroplasticity and motor recovery. While traditional rehabilitation may involve passive repetitions, modern robotic exoskeletons incorporate motion intention estimation to make these exercises more adaptive and patient-driven. Motion intention estimation allows the exoskeleton to synchronize movement assistance with the patient’s voluntary effort, ensuring that therapy remains engaging and aligned with neuromuscular recovery. The following subsections describe how this principle applies to different rehabilitation therapies [11,30,31].

A fundamental aspect of rehabilitation exoskeletons is their ability to synchronize with the user’s motor intention to enhance recovery. Motion intention estimation allows the system to detect voluntary or residual movements, adapting assistance accordingly. Meanwhile, control strategies ensure movement execution aligns with therapeutic goals, whether by providing predefined trajectories, assisting voluntary effort, or coordinating bilateral limb actions. For rehabilitation applications, robotic devices assist physical therapists in administering rehabilitative exercises. These devices typically support three types of therapies based on established rehabilitation protocols [32,33,34].

1. Passive Therapy: This form of therapy does not require any effort from the patient during rehabilitation movements. It is typically used in the early stages of rehabilitation or when there is no voluntary response in the affected joint or limb (e.g., post-stroke) [33,34]. The therapy involves repeatedly moving the joint in specific trajectories, effectively reducing spasms and preventing muscle atrophy in the involved joint [35].
2. Active Therapy: This type of therapy allows patients to perform some voluntary movements in their affected joints, though they may still lack strength or efficiency. It can be divided into active–assistive therapy and active–resistive therapy. In active–assistive therapy, the patient attempts to move the affected limb voluntarily. At the same time, the robotic device provides external force assistance [36], improving the range of motion of the joint [34]. In active–resistive therapy, the patient tries to perform voluntary movements while the robotic device generates resistance [35], helping to gradually increase muscle strength in the treated joint [34].
3. Bilateral Therapy: In this therapy, the affected limb mirrors the movements of the functional limb (mirror therapy). Some exoskeleton systems support this type of therapy, which is commonly applied in stroke recovery [34].

Traditionally, robotic assistance and rehabilitation devices have been constructed with rigid structural elements positioned parallel to the user’s limbs to facilitate joint assistance through load transmission [37,38]. Although these devices can provide high torque and achieve good trajectory tracking, their rigid structure presents several disadvantages. These devices tend to be heavy and bulky, which increases the system’s inertia and the user’s metabolic cost, as well as makes transport and operation more challenging. Additionally, the joint connecting the device’s rigid elements must be precisely aligned with the user’s joint to provide effective assistance. This alignment is difficult because, in some human joints, the rotation center changes within the range of motion [39]. This misalignment often results in discomfort and reduced performance in assistance and rehabilitation tasks [26,40]. These issues have prompted researchers to explore new solutions, such as exoskeletons based on soft robotics.

### 3.2. Soft Robotics Exoskeletons

Robotic systems can be classified based on their structures and ability to deform. Under this classification, robots can be either rigid or soft. Unlike rigid exoskeletons, where force transmission is more direct, soft exoskeletons require control models that compensate for material deformation and variability in actuator response. This translates into the need for advanced strategies, such as adaptive impedance control, which allows system stiffness to be adjusted in real-time based on EMG signals and user interaction. Soft robots are constructed with easily deformable materials and structures with minimal resistance to compressive forces, enabling them to respond better when encountering obstacles in unstructured environments [41].

The advantages of soft robotics have driven research toward developing biomimetic systems that mimic natural structures. These systems aim to be elastic, deformable, and capable of navigating confined spaces without suffering damage from high pressures or stress concentrations [42,43,44,45]. Moreover, besides their soft qualities, these robots also strive for sufficient adaptability to work safely alongside humans and other soft structures in their environment [46].

Soft robotics has emerged as a promising trend in wearable robotics, particularly in assistance and rehabilitation. Its ability to quickly adapt to the contours and movements of human joints allows for greater comfort and performance in these tasks [8,9,10,11,47].

**Figure 2:**
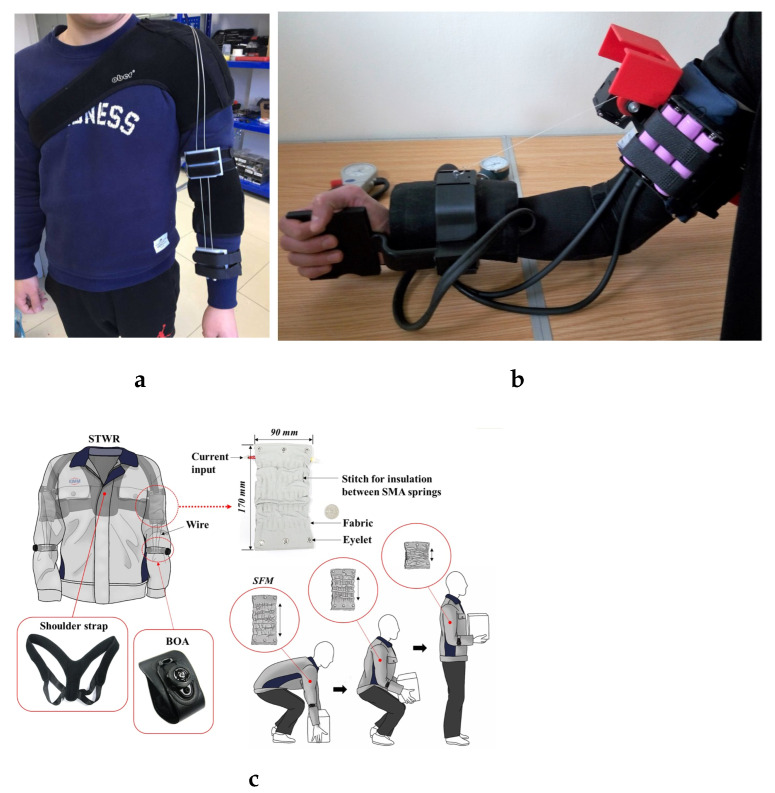
Soft robotics rehabilitation and assistance exoskeletons. (**a**) Bowden cable-driven soft exoskeleton [51]. (**b**) CADEL (CAble Driven device for Elbow assistance) soft exoskeleton [50]. (**c**) Suit-type shape-memory alloy (SMA) soft exoskeleton [30].

Given these advantages, the past decade has seen growing research interest in soft robotic exoskeletons. Various soft robotic exoskeletons have been developed for different applications, including augmentation, assistance, and rehabilitation. These devices aim to provide a smooth interface at the joint level to leverage the benefits of their soft structures, although they may include rigid components elsewhere in the design. Most proposed exoskeletons use pneumatic actuators [11,48,49] or cable-driven systems [31,50,51,52,53] to generate movement, though other actuation methods like shape-memory alloy actuators [30,54,55] have also been explored. Figure 2 illustrates some examples of developed soft robotic exoskeletons.

Other papers have focused on soft robotics exoskeletons; for instance, Shi and Yongjun et al. [37] discuss the current development status of soft wearable robots for various human joints. Bardi and Gandolla et al. [26] provide an extensive list of upper limb assistance robotic devices. Xiloyannis and Alicea et al. [56] studied different modes of actuation, physical human–robot interfaces, and motion intention algorithms. Thalman and Artemiadis et al. [57] present a meta-analysis of findings on various actuation methods for wearable robots. Lastly, Bogue [27] explores multiple exoskeletons designed for industrial applications.

### 3.3. Myoelectric Control

Surface electromyography (sEMG) is a technique used to study the electrical activity in muscles during contraction, whether during dynamic movement or isometric holds. This electrical activity, resulting from the contraction of muscle fibers, is captured through electrodes placed superficially on the muscle being analyzed [21,58,59,60].

The EMG signal has been extensively studied over the years because it can be related to important variables such as muscle activation levels and the force generated by muscles [60]. Recently, interest in studying these signals has grown due to their potential for creating human–machine interfaces that enable devices such as exoskeletons for assistance and rehabilitation to be operated intuitively and efficiently [12,13,21].

**Figure 3:**
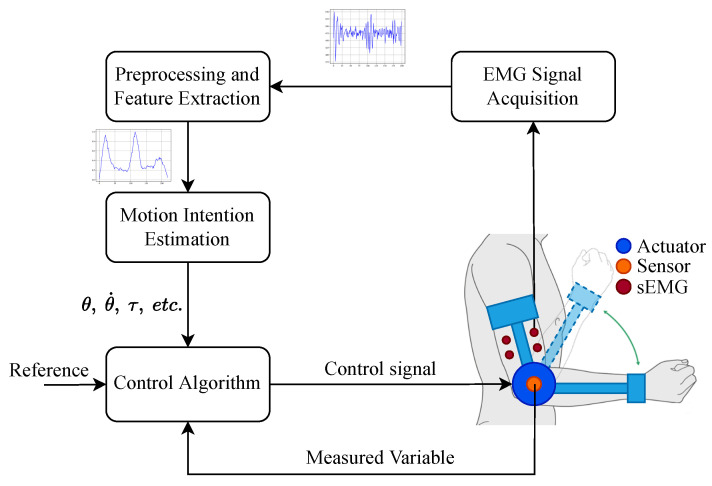
Myoelectric control scheme.

Using EMG as control signals for exoskeletons or prostheses is known as myoelectric control. Typically, a myoelectric control loop uses the EMG signal to estimate dynamic variables such as joint position, speed, torque, and stiffness. These variables either serve as the set point for the control system or allow the robotic exoskeleton to adapt and synchronize with the user’s movement during assistance or rehabilitation tasks. Figure 3 depicts a classical myoelectric control scheme and its various components.

**Figure 4:**
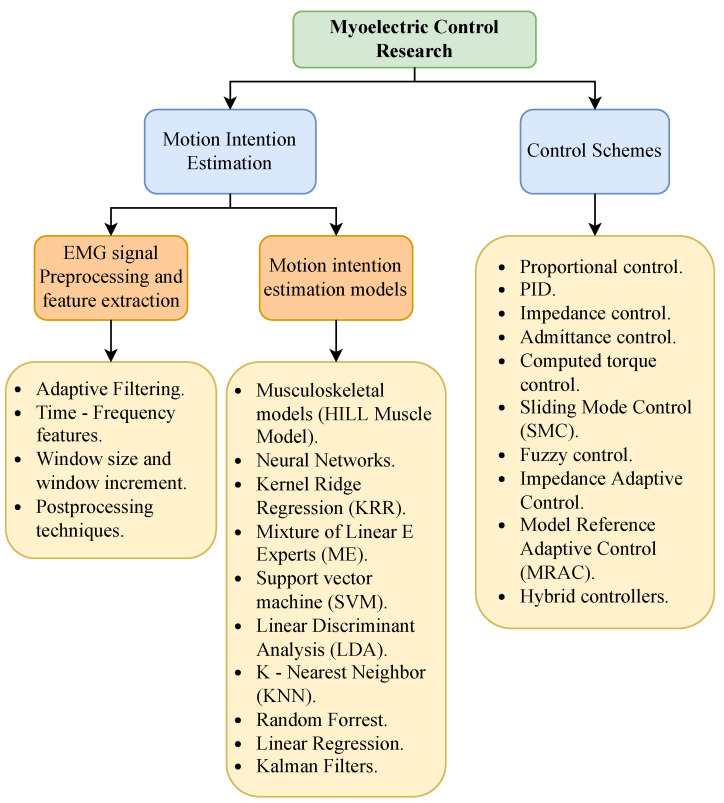
Myoelectric control research.

In recent decades, there has been growing interest in researching the EMG signal as a control mechanism. The advancements in this field can be divided into two primary research areas. The first area focuses on developing motion intention estimation algorithms for classification and regression tasks. Some of this research centers on creating new preprocessing and feature extraction techniques to enhance the performance of these algorithms. The second area concentrates on developing control schemes that adjust to the user’s movements based on the variables predicted by the motion intention estimation algorithms. Figure 4 provides an overview of these two research areas and the progress made in this field.

The following sections will describe in more detail each of the stages present in a myoelectric control system, along with the research found in the state-of-the-art.

#### 3.3.1. sEMG Acquisition

The process of acquiring the EMG signal involves placing a pair of electrodes on the skin above the muscle. These electrodes are connected to a differential operational amplifier, which allows the signal to be read by an analog-to-digital converter (ADC) for further analysis using computational tools. The effectiveness of this acquisition method depends on the electrodes’ location, their position relative to muscle fibers, and the distance between them. More advanced acquisition methods utilizing 1-D and 2-D electrode arrays, which enable the mapping of muscular activity in specific body areas, have also been researched and are commonly referred to as muscle synergies. For more detailed insights into the EMG acquisition process, readers can refer to the books by Merletti and Farina, “Surface Electromyography: Physiology, Engineering and Applications” [21], and by Criswell, “CRAM’s Introduction to Surface Electromyography” [58].

#### 3.3.2. sEMG Preprocessing and Feature Extraction

To use the EMG signal as a control input, the signal must undergo some processing. This is a crucial step in every myoelectric control scheme because the raw EMG signal’s instantaneous value is not particularly useful due to its random nature [61]. This stage is typically known as the EMG signal’s preprocessing and feature extraction phases. Generally, several steps are involved in these phases.

The initial step is to filter out noise in the raw EMG signal, eliminating frequency components not part of the EMG signal, such as additive noise, the DC offset component, artifacts, and power-line interference. It is common to use a band-pass filter with cutoff frequencies $f_{min}=10\;\mathrm{Hz}$ and $f_{max}=500\;\mathrm{Hz}$, covering the EMG signal’s bandwidth [21,58,60,62]. Some acquisition systems also include a notch filter with a stopband frequency of $f_{sb}=60\;\mathrm{Hz}$ to reduce power-line noise, although this filter is not always recommended.

The signal typically undergoes demodulation once the raw EMG is noise-free and without DC offset. This process usually involves taking the absolute value (also known as full-wave rectification) or the square of each sample. Although demodulation techniques such as full-wave rectification are widely used, some studies criticize their use since, being a nonlinear transformation, they may introduce distortion to the EMG signal [21].

The next step is partitioning the EMG signal into multiple windows to apply a feature extraction technique. Window segmentation of the signal before feature extraction is critical because the performance of motion intention algorithms depends on appropriately selecting window characteristics [13,21,61,62].

**Figure 5:**
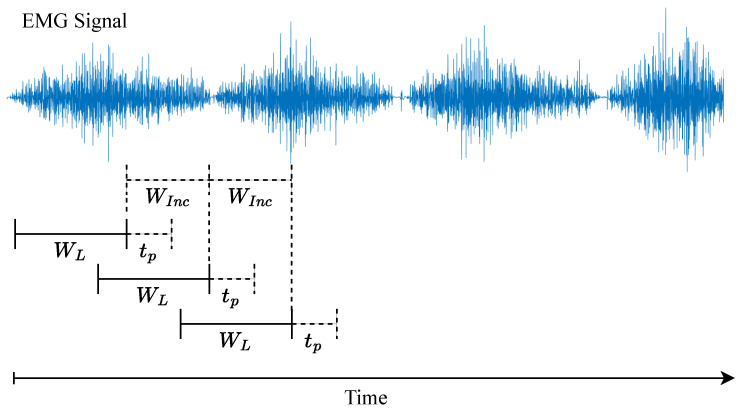
Sliding window technique. $W_{L}$ corresponds to the window length, $W_{Inc}$ to the window increment, and $t_{p}$ to the processing time of the EMG motion intention estimation algorithm.

The overlapping window or sliding window technique is used to segment the EMG signal. This technique divides the EMG signal into windows with a length $W_{L}$. Once a window is captured, the next window is taken after a certain time or window increment $W_{Inc}$ has passed [61,62,63]. Figure 5 illustrates how the sliding window technique works.

Typically, the window increment $W_{Inc}$ is chosen to be shorter than the window length $W_{L}$ and ideally should match the algorithm processing time $t_{p}$ to achieve the maximum window density for processing [61] and to minimize actuation delay. Larger window lengths allow for better extraction of the EMG signal’s characteristics and tend to provide greater accuracy in motion intention estimation models. However, longer windows can also increase the system’s actuation delay time [62,63]. In practice, window lengths commonly range between 100 and 200 ms [13,21,61], while window increment values can vary significantly across studies.

Once the raw EMG signal is preprocessed following the steps above, a feature extraction process can be performed to obtain valuable information about the signal’s amplitude and power. These characteristics can provide insights into muscle contraction intensity and the signal’s frequency content, which can be useful for determining muscle fatigue, among other things. These characteristics are generally classified into three domains [13,21,62]. Table 1 summarizes the different features of the sEMG signal.

##### Time-Domain Features

Time-domain features focus on analyzing the amplitude of the EMG signal. These features represent the energy of the EMG signal, the level of muscle activation, the duration of muscle contraction, and the relationship between signal intensity and force. Some features also relate to frequency properties, such as the Zero Crossings feature. These features are widely used because they are simple and computationally inexpensive, and they provide a direct relationship between the EMG signal and physiological variables such as muscle strength. However, they are sensitive to noise and amplitude cancellation [13].

##### Frequency-Domain Features

Frequency domain features offer insights into the rate of muscle activation, enabling the description of non-stationary signals and the analysis of the EMG signal’s power and energy. These features are particularly valuable for detecting muscle fatigue; however, they tend to be more computationally demanding, have lower temporal resolution, and may exhibit high variance [13,21].

##### Time–Frequency-Domain Features

Time–frequency-domain features enable the identification of transient and steady-state patterns during dynamic muscle contractions. These features are particularly useful for analyzing patterns in high-dimensional domains, such as when there is a high density of EMG acquisition channels. They offer the advantages of both time-domain and frequency-domain features. However, they are generally complex and computationally expensive due to the high number of parameters involved, resulting from the large number of EMG channels [13].

These three types of features can be used to extract valuable information from the EMG signal, regardless of whether the acquisition is achieved using a single channel or multiple EMG channels, also known as high-density EMG or Muscle Synergies analysis [13,21]. The latter method allows for a more comprehensive mapping of muscle activity compared to single-channel techniques. Although the previously mentioned features are the most commonly used for extracting information from the EMG signal, other feature extraction and preprocessing techniques exist [64]. For example, Hofmann et al. proposed a recursive nonlinear estimator of sEMG amplitude based on Bayesian filtering [65]. This adaptive filter enhances performance in simultaneous proportional control tasks. Additionally, Stachaczyk et al. suggested adaptive filtering techniques for high-density EMG based on reinforced formulation [66]. Other studies have proposed combining features from the three domains for improved performance [67].

#### 3.3.3. sEMG Motion Intention Estimation

Given the primary objective of robotic assistance and rehabilitation exoskeletons, which is to gradually restore a patient’s motor function or assist in various tasks, myoelectric control plays an important role by considering the patient’s motion intention during exoskeleton operation. To achieve this, an interface must be established with the patient’s nervous system (human–machine interface); in the case of myoelectric control, this interface occurs at the muscular level.

Generally, myoelectric control schemes or human–machine interfaces based on EMG signals aim to generate a control signal that activates the exoskeleton, ensuring it works in sync with the user. There are mainly two approaches to generating this control signal using EMG signals [21]. The first approach involves model-based interfaces, which use specific musculoskeletal models tailored to each user. The second approach consists of data-driven interfaces that rely on machine learning models. It is important to note that these interfaces utilize the processed EMG signal, meaning the input to the interface or model is the EMG signal after preprocessing and feature extraction.

##### Model-Based Interfaces: EMG Musculoskeletal Models

Model-based interfaces are designed to calculate various internal physiological variables dependent on the EMG signal that cannot be directly derived from the EMG signal analysis or human kinematics. These models have an advantage over data-driven models as they provide a deeper human–machine interface by considering specific patient parameters operating the exoskeleton. Some variables represented by these models include muscle strength, joint angle, and joint stiffness [68,69,70]. A crucial consideration for these interfaces is that they are subject-specific and require constant calibration and scaling of their parameters to be effective across different subjects [21,71].

Multiple studies have proposed various musculoskeletal models using EMG signals as input. One classical model is the HILL-type muscle model [59], used to determine muscle force during movement. Multiple studies have implemented EMG musculoskeletal interfaces based on the HILL muscle model [72,73,74]. Other researchers have proposed flexible models solely based on EMG signals optimized via genetic algorithms [75]; neuromusculoskeletal models with user-based parameter scaling [76]; online calibrated EMG-informed neuromusculoskeletal models [77]; patient-specific models based on neural surrogates [78]; and linear and genetic optimization techniques for model parameter selection [70].

##### Data-Driven Interfaces: Machine Learning Motion Intention Algorithms

Data-driven interfaces primarily rely on machine learning models for motion intention estimation. Generally, there are two main approaches to motion intention estimation using machine learning: classification and regression [21].

The first type of data-driven interfaces involves classification or clustering machine learning models that aim to identify distinctive patterns in the EMG signal within a given time interval. Each EMG window is associated with a defined class or task based on these identified patterns. This association is achieved by mapping features extracted from the EMG signal to different classes using a labeled dataset, where each EMG recording interval corresponds to a class (supervised learning). Some machine learning models that do not require labeled data (unsupervised learning or clustering) can also be used.

Classification models are typically used in devices that need to perform a range of tasks rather than a single movement. Various models have been proposed for classification interfaces, including neural networks [79,80], Support Vector Machines (SVM) [80,81], Linear Discriminant Analysis (LDA) for motion intention classification [79,81,82,83,84,85] and isometric torque classification [86], K-Nearest Neighbors [87], Naive Bayes, decision trees [80], and ensemble methods like Random Forest [88] and AdaBoost [89].

The second type of data-driven interface involves regression models, where the EMG signal is mapped to a continuous signal rather than a series of discrete classes. These interfaces are helpful for proportional control of exoskeletons and are generally based on time-varying features of the EMG signal, such as its amplitude. Regression models can learn their parameters like classification models through supervised or unsupervised learning methods.

Regression models have also seen significant development in the literature. Commonly explored regression models include Linear Regression using antagonist muscles [90], Multivariate Regression [12], Kernel Ridge Regression (KRR) [91], Neural Networks [91,92], nonlinear EMG to muscle activation mapping (ACT) [93], and Kalman filters based on neural networks [94] and adaptive models [10]. One strategy that stands out for its ability to perform unsupervised regression (without labeled data) is Non-Negative Matrix Factorization (NNMF) [14,21].

Table 2 presents a comparative analysis for sEMG motion intention estimation approaches.

### 3.4. Control Strategies

The control strategy is a crucial component of a myoelectric control scheme. Generally, the goal of a control algorithm is to guide the system to a desired state based on specific performance criteria, such as minimizing tracking error, reducing steady-state error, decreasing system oscillations, stabilizing the system, and rejecting disturbances. To meet these requirements, the control algorithm uses both the measured variable and the setpoint (or reference) to determine a control signal that drives the system toward the desired state, a method commonly known as feedback control [95,96].

Control strategies can be categorized into different approaches based on their performance requirements. Classical or typical control ensures certain transient or steady-state behaviors [95,96]. Optimal control seeks to find the best strategies for controlling a dynamic system, usually based on minimization objectives [97]. Robust control addresses model uncertainties, which are characterized as perturbations of a nominal system model, with the goal of meeting performance requirements despite these uncertainties [96]. Adaptive control involves parameterizing the system’s uncertainty in terms of unknown parameters, which are learned online during system operation using feedback from the measured variable [96,98,99]. Some strategies combine different control approaches to enhance the control algorithm’s performance.

A variety of algorithms have been proposed for controlling upper limb exoskeletons. Several researchers have suggested model-based controllers for rehabilitation exoskeletons [100] and augmentation exoskeletons [101]. Others, like Copaci, have combined these model-based methods with motion intention estimation algorithms using EMG signals [55]. However, this approach presents the challenge of requiring a dynamic model of the exoskeleton, which is generally difficult to obtain given the complexity of these systems, the interaction with the user, and parameter uncertainties. This drawback is particularly pronounced in soft robotic devices.

Due to the limitations of model-based designs, many researchers propose adaptive controllers that do not need knowledge of the system dynamics. Adaptive controllers have been utilized across various domains and have demonstrated fast convergence, accurate trajectory tracking, and the capability to handle parameter uncertainties [102,103,104,105,106]. Numerous researchers have also suggested voluntary control schemes based on sEMG signals. Among these, the use of proportional controllers that do not require joint torque estimation stands out [107]; further, voluntary control of variable stiffness exoskeletons [108], adaptive impedance and admittance controllers that utilize EMG signals for both motion intention and joint stiffness estimation [109,110], hybrid impedance/admittance controllers [111], controllers based on fuzzy model estimation and sEMG motion intention prediction [112,113,114], and human-like control based on adaptive feedback with dynamic compensation also stand out [115,116].

Although the controllers mentioned above facilitate voluntary exoskeleton control, they require user-dependent calibration procedures and a certain level of EMG activation that some patients may lack. Considering this, other researchers have proposed controllers designed solely for passive assistance. One commonly used scheme is the adaptive impedance controller [84,117,118], which predicts joint stiffness using the sEMG signal so that the controller parameters align with joint dynamics. Additionally, other controllers such as adaptive controllers for periodic movements based on Dynamical Movement Primitives [119]; adaptive sliding mode controllers [120,121]; adaptive controllers based on neural networks for estimating exoskeleton dynamics [106,122]; Model Reference Adaptive Controllers with an adaptive Kalman Filter [123]; and Model Reference Adaptive Impedance Controllers [124,125,126] have also been proposed. Despite their significant advantages in not requiring knowledge of the system dynamics and adapting to the user, these controllers have drawbacks, including high computational cost, the need for a learning phase or calibration procedure, and potential unwanted behaviors during the learning process [127,128].

As observed, most proposed controllers for exoskeletons are based on adaptive strategies. This is because devices that interact with humans and need to adjust to the biomechanical behavior of a limb often have numerous uncertainties in their models or are complex to model. Therefore, control strategies that are not model-dependent and can adapt to different users and operating conditions show great promise in the field of rehabilitation and assistive exoskeletons.

Despite the various control strategies proposed, each has its drawbacks and disadvantages. For non-adaptive controllers like proportional, impedance, or admittance controllers, understanding the relevant system models is necessary, which is sometimes not feasible. Conversely, complex controllers with multiple parameters, like adaptive strategies, can be challenging to ensure robustness and stability, potentially leading to unwanted system behaviors. Additionally, adaptive strategies often require a learning or calibration process and are computationally intensive [127,128].

## 4. Challenges and Future Directions

As mentioned earlier in this review, myoelectric control has shown promise due to its ability to provide an intuitive control interface that allows the exoskeleton to adapt to the user’s movements. However, there are still challenges in myoelectric control schemes that hinder their implementation in exoskeletons and limit their commercial application. The primary challenge in myoelectric control schemes is the need to actuate the exoskeleton in synchrony with the user’s movements to provide effective rehabilitation therapy or assistance [12,13]. This primary challenge arises from three specific difficulties.

1. Motion Intention Estimation Algorithms: There are still significant challenges in EMG-based motion intention estimation algorithms, a core component of myoelectric control schemes. These challenges include performance, repeatability, computational cost, and computational delay [129,130]. They primarily stem from two factors. The first is the difficulties present in the sEMG acquisition process, which depends on multiple variables that are hard to reproduce across different acquisition sessions, leading to variations in the acquired EMG signal, as mentioned in Section 3.3.1. The second factor is that the EMG signal morphology changes over time due to phenomena like muscle fatigue [13,24]. These factors require motion intention algorithms to be subject-specific and constantly calibrated to perform well.
2. Modeling and Control of Soft Robotic Systems: The recent trend of soft robotics introduces major challenges regarding controlling exoskeletons based on soft systems. This challenge is also due to two primary factors. The first is the difficulty in modeling soft systems because of parameter uncertainties and the possibility of having infinite degrees of freedom, making it hard to implement traditional model-based control strategies for these systems. The second factor is that, given the deformable and soft nature of these devices, traditional position sensors are often unsuitable, making it difficult to measure the control variable in soft systems. This issue is evident in soft exoskeletons, where the joint position should be measured without aligning the joint axis with the sensor to avoid user discomfort [131,132,133,134].
3. Validation and Evaluation of Myoelectric Controllers: Although many studies have proposed EMG motion intention estimation algorithms for myoelectric control schemes, most of these investigations have been limited to validating the algorithms using only static data and simulations. However, it has been found that models that perform well on static data do not necessarily exhibit good performance during real-time implementations [22,135]. This causes uncertainties regarding whether EMG motion intention algorithms are suitable for online operation.

To address the challenges in myoelectric control systems for rehabilitation and assistance exoskeletons, several promising future directions have emerged. One significant area of focus is the development of data-driven motion intention estimation algorithms based on machine learning models. These algorithms leverage large datasets to train models that can accurately predict user intentions in real time. By utilizing advanced machine learning techniques such as deep learning and reinforcement learning, these data-driven approaches can enhance the adaptability and precision of myoelectric control schemes, making them more effective for a diverse range of users [136]. One of the main challenges in myoelectric control for soft exoskeletons is the difficulty in accurately predicting the response of the soft material to an EMG signal. Unlike rigid exoskeletons, where the relationship between muscle activation and movement is more direct, in soft systems, the actuator response depends not only on the control signal but also on the elasticity, friction, and response time of the material. This requires exploring new machine learning strategies and hybrid models that combine model-based control with adaptive learning techniques. Recent studies have demonstrated the potential of these techniques to improve control accuracy and efficiency in rehabilitation devices [136,137,138].

Another promising direction is the implementation of model-free control strategies based on adaptive control systems and machine learning models like reinforcement learning. These strategies do not rely on precise knowledge of the system dynamics, making them well suited for the complex and variable nature of human–exoskeleton interactions. Model-free adaptive control methods have shown great potential in ensuring closed-loop system stability and handling parameter uncertainties [139,140,141,142,143]. By integrating reinforcement learning algorithms, these control strategies can continuously learn and adapt to the user’s movements, providing more personalized and effective assistance [144].

Finally, the real-time validation of myoelectric controllers is crucial for ensuring their practical applicability and reliability. Conducting real-time evaluations and robustness tests can help identify and address potential performance, repeatability, and computational delay issues. Recent research has highlighted the importance of validating myoelectric control systems in dynamic and real-world scenarios to ensure their effectiveness in practical applications. Researchers can refine and optimize myoelectric control algorithms by incorporating real-time validation processes, ultimately enhancing their performance and facilitating their transition from experimental settings to real-world use [145].

These future directions are promising for advancing myoelectric control systems and overcoming the current challenges in rehabilitation and assistance exoskeletons. Continued research and innovation in these areas are essential for unlocking the full potential of these technologies [145].

## 5. Key Findings

In this comprehensive review, several critical insights have emerged regarding the advancements, challenges, and future directions of soft robotic exoskeletons and myoelectric control strategies for rehabilitation and assistance. The following key findings highlight the primary contributions and observations from the reviewed literature.

**Figure 6:**
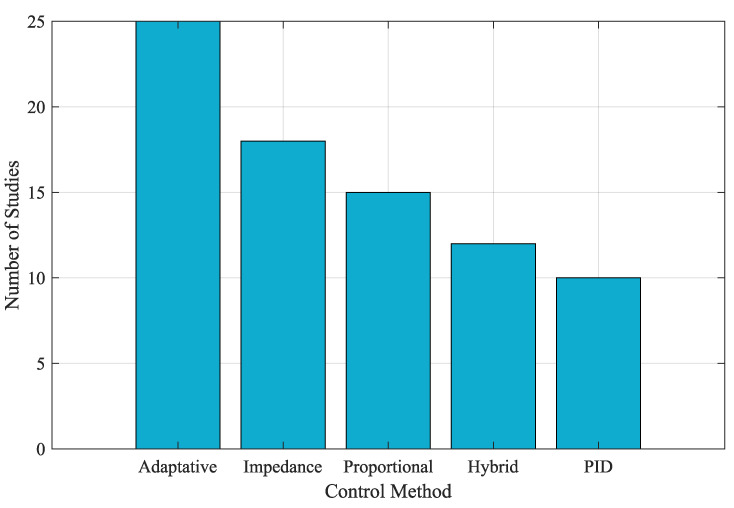
Comparison of control strategies in exoskeletons.

**Figure 7:**
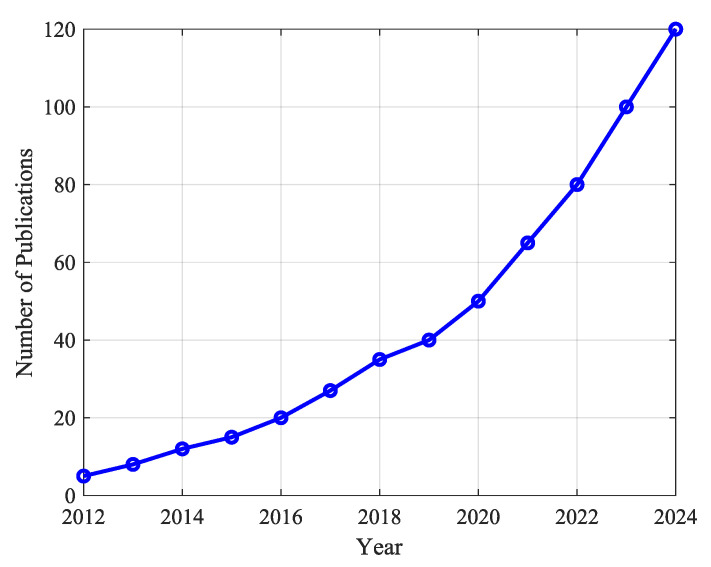
Trends in soft robotics exoskeleton publications from 2012 to 2024.

**Figure 8:**
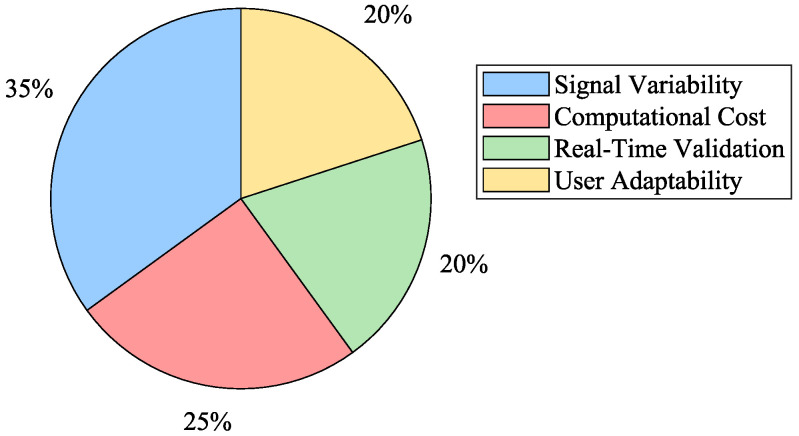
Challenges in myoelectric control.

1. Innovative Design of Soft Robotics Exoskeletons
  Integrating soft materials in robotic exoskeletons has revolutionized the field by enhancing adaptability and comfort. Soft robotic exoskeletons have demonstrated significant potential in conforming to human body contours and providing better assistance in rehabilitation and daily activities. Their ability to provide effective rehabilitation therapies and help in movement assistance tasks without causing discomfort to the user marks a significant advancement over traditional rigid exoskeletons.
2. Integration of Soft Actuation Methods
  The development of various actuation methods, including pneumatic actuators, cable-driven systems, and shape-memory alloys, has expanded the capabilities of soft robotic exoskeletons. These advanced actuation techniques contribute to the flexibility and efficiency of the devices, enabling them to perform a wide range of motions and tasks. The integration of these methods supports the creation of more sophisticated and functional exoskeletons tailored to specific rehabilitation and assistance needs.
3. Comparison of Control Strategies in Exoskeletons
  Control strategies play a crucial role in exoskeleton design. As shown in Figure 6, adaptive and impedance-based control methods are the most widely used due to their ability to handle uncertainties and human–exoskeleton interactions. These approaches enable more flexible and robust control schemes, particularly in applications requiring real-time adaptability.
4. Effectiveness of Myoelectric Control
  Myoelectric control strategies have shown promising results in enabling the intuitive control of exoskeletons. These strategies allow exoskeletons to adapt and synchronize with the user’s movements, facilitating more effective rehabilitation and assistance. Using sEMG signals to estimate dynamic variables such as joint position, speed, and torque has proven feasible for achieving seamless human–machine interaction.
  While myoelectric control has shown significant promise for facilitating intuitive interactions in rehabilitation and assistance exoskeletons, several limitations challenge its commercial application. As mentioned, challenges in the sEMG signal acquisition process, such as electrode placement, skin impedance, and muscle fatigue, can compromise signal consistency and accuracy. This inconsistency is transferred to the controller via the myoelectric interface, often causing delayed or erroneous interpretations of the user’s motion intent, which can be particularly problematic in clinical settings where precise timing and movement coordination are essential. Furthermore, in patients with neuromuscular impairments, the diminished or irregular electrical activity of muscles can further degrade control performance, thus limiting the effectiveness of the interface. This is one of the reasons why myoelectric control interfaces are, for the most part, still limited to controlled testing environments.
  Additionally, current myoelectric interfaces face challenges related to environmental interference and morphological changes in the signal, which complicate signal processing and may require extensive calibration. Such recalibrations can be time-consuming and require frequent adjustments to accommodate day-to-day physiological changes or activity-induced variations in the sEMG signal. To address these limitations, future research will integrate complementary sensing modalities, such as inertial measurement units (IMUs) or mechanomyography (MMG), and develop advanced signal processing algorithms that enhance noise reduction and improve interpretability. These approaches could produce a more robust, reliable, and user-adaptive control system that optimizes the performance of rehabilitation and assistance exoskeletons.
5. Machine Learning Models for Motion Intention Estimation
  Data-driven methods based on machine learning techniques are gaining traction in motion intention estimation. These methods leverage large datasets to train models that can accurately predict user intentions in real time. By utilizing machine learning algorithms, these data-driven approaches enhance the adaptability and precision of myoelectric control schemes, making them more practical for a diverse range of users without requiring long calibration processes.
6. Trends in Soft Robotics Exoskeleton Publications
  The increasing research interest in soft robotic exoskeletons is evident in the publication trends over the last decade, as depicted in Figure 7. The number of publications in this area has grown significantly since 2012, demonstrating a strong research focus on improving adaptability and usability in rehabilitation and assistive robotics.
7. Challenges in Motion Intention Estimation
  Despite the advances in myoelectric control, significant challenges remain in motion intention estimation algorithms. Performance, repeatability, computational cost, and delay are critical issues that must be addressed. The variability in sEMG signal acquisition and the changes in signal morphology due to muscle fatigue necessitate ongoing calibration and adaptation of motion intention algorithms to ensure their accuracy and reliability. Figure 8 summarizes the primary obstacles encountered in myoelectric control implementation.
8. Adaptive Control Strategies
  Adaptive controllers have emerged as a robust solution for dealing with the complexities and uncertainties inherent in exoskeleton dynamics. These controllers, which do not require precise knowledge of the system’s dynamics, have shown fast convergence, accurate trajectory tracking, and effective parameter handling. The application of adaptive control strategies, particularly in soft robotic exoskeletons, holds great promise for enhancing performance and user experience. This is due to their ability to overcome the challenge of modeling and parameter tuning for controlling soft robotic exoskeletons. These strategies allow the controllers to adapt to the nonlinear, variable nature of soft actuators, and they can adjust to different users with varying anatomic and kinematic parameters; in particular, this is important as it facilitates the operation of the exoskeleton.
  Despite their advantages, adaptive control strategies face some important drawbacks. As mentioned, they are generally computationally expensive, which might limit their implementation in portable devices with limited hardware capabilities. They often require extended calibration and learning phases, which can delay deployment and may introduce transient instabilities. Moreover, their inherent sensitivity to parameter variations can lead to performance degradation over time, further challenging the consistency essential for safe and effective operation.
  To address these drawbacks, several strategies could be implemented. First, algorithm optimization techniques could be used to reduce computational overhead. Second, using model-free learning strategies, for example, based on reinforcement learning, can minimize reliance on precise system dynamics while maintaining adaptability. Third, implementing pre-trained models that integrate generalized motion data could reduce calibration time. Pairing this with user-specific fine-tuning could enhance system reliability and shorten deployment phases. Lastly, continuous real-time testing during usage could facilitate automatic recalibration, enhancing the device’s adaptability to parameter changes.

While this review offers an extensive examination of myoelectric control systems for rehabilitation and assistance exoskeletons and integrating these devices with soft robotics, certain limitations should be noted. Primarily, the study is confined to peer-reviewed articles from databases like SCOPUS and Web of Science. This restriction may exclude pertinent insights from new research platforms, technical reports, patents, and industry advancements. The continuous evolution of soft robotics exoskeletons and myoelectric control systems presents a challenge in keeping the synthesis up-to-date, as innovations in technology and methods often surpass the review’s timeline.

Moreover, although this review identifies key challenges in integrating myoelectric control systems with soft robotics exoskeletons, it does not delve deeply into the ethical, economic, or deployment considerations that might affect commercial implementation in industrial or biomedical settings. Future research should prioritize experimental validation, interdisciplinary evaluations, and comprehensive technological forecasting to enhance the relevance and effectiveness of soft exoskeletons with myoelectric control schemes.

## 6. Conclusions

The field of soft robotic exoskeletons for rehabilitation and assistance is evolving rapidly, driven by advancements in myoelectric control and the integration of soft materials that enhance user comfort and adaptability. Unlike traditional rigid exoskeletons, soft robotic systems offer a more natural interaction with the user, requiring novel control strategies to compensate for material deformability and ensure precise assistance. This review has highlighted key trends in myoelectric control, including machine learning-based motion intention estimation, model-free adaptive control methods, and real-time validation strategies.

Despite these advancements, critical challenges remain, particularly regarding the variability of sEMG signals, real-time computational demands, and the difficulty of achieving stable and responsive control in soft robotic architectures. Addressing these limitations is essential for translating these technologies into clinically viable rehabilitation solutions. A key observation from our review is the necessity of integrating robust adaptive control frameworks that can dynamically adjust assistance based on user needs, preventing over-reliance while promoting motor recovery. Additionally, future research should explore hybrid approaches that combine myoelectric control with alternative sensing modalities to improve signal robustness and reliability.

Beyond summarizing the state-of-the-art, this review provides a critical perspective on myoelectric control’s current limitations and future directions in soft exoskeletons. By refining control methodologies and improving real-time adaptability, these technologies can significantly enhance rehabilitation outcomes, paving the way for more effective, personalized, and clinically deployable assistive solutions.

## Figures and Tables

**Table 1:** Fabrication methods in soft robotics.

Domain	Description	Features
Time-Domain Features	Focus on analyzing the amplitude of the EMG signal. Simple, computationally inexpensive, but sensitive to noise.	Root Mean Square (RMS). Mean Absolute Value (MAV). Waveform Length (WL). Linear Envelope (LE). Zero Crossing (ZC). Slope Sign Changes.
Frequency-Domain Features	Focus on the rate of muscle activation. Computationally expensive and with high variance.	Power Spectral Moments. Power Spectral Density (PSD). Median Frequency (MDF). Mean Frequency (MNF). Short-Time Fourier Transform.
Time–Frequency-Domain Features	Allow identification of both transient and steady-state patterns in the sEMG signal. Computationally expensive.	Wavelet Packet Transform. Discrete Wavelet Transform.

**Table 2:** Comparative analysis of motion intention estimation strategies.

Strategy	Pros	Cons	Real-World Constraints/ Implementation Considerations
Model-Based Interfaces	Provide deep physiological insights. Tailored to subject-specific parameters.	Require detailed musculoskeletal modeling. Demand frequent calibration and are sensitive to parameter uncertainties.	Precise sensor placement is critical. Challenging to adapt to dynamic user conditions and hardware limitations.
Data-Driven Interfaces	Flexible and adaptable through machine learning. Leverage large datasets for training.	High computational cost. Performance heavily depends on signal quality and training data.	Requires robust, real-time processing capabilities. May necessitate frequent retraining to accommodate user variability.

## Data Availability

The original contributions presented in the study are included in the article; further inquiries can be directed to the corresponding author(s).
